# In Vivo Study of Aerosol, Droplets and Splatter Reduction in Dentistry

**DOI:** 10.3390/v13101928

**Published:** 2021-09-25

**Authors:** Naeemah Noordien, Suné Mulder-van Staden, Riaan Mulder

**Affiliations:** 1 Paediatric Dentistry, The University of the Western Cape, Cape Town 7530, South Africa; nnoordien@uwc.ac.za; 2 Oral Medicine, Periodontology and Implantology Department, The University of the Western Cape, Cape Town 7530, South Africa; 3 Restorative Dentistry, The University of the Western Cape, Cape Town 7530, South Africa; rmulder@uwc.ac.za

**Keywords:** SARS-CoV-2, dental aerosol-generating procedures, extra-oral suction, high-volume evacuation, low-volume saliva ejector, splatter, aerosol

## Abstract

Oral health care workers (OHCW) are exposed to pathogenic microorganisms during dental aerosol-generating procedures. Technologies aimed at the reduction of aerosol, droplets and splatter are essential. This in vivo study assessed aerosol, droplet and splatter contamination in a simulated clinical scenario. The coolant of the high-speed air turbine was colored with red concentrate. The red aerosol, droplets and splatter contamination on the wrists of the OHCW and chests of the OHCW/volunteer protective gowns, were assessed and quantified in cm^2^. The efficacy of various evacuation strategies was assessed: low-volume saliva ejector (LV) alone, high-volume evacuator (HV) plus LV and an extra-oral dental aerosol suction device (DASD) plus LV. The Kruskal–Wallis rank-sum test for multiple independent samples with a post-hoc test was used. No significant difference between the LV alone compared to the HV plus LV was demonstrated (*p* = 0.372059). The DASD combined with LV resulted in a 62% reduction of contamination of the OHCW. The HV plus LV reduced contamination by 53% compared to LV alone (*p* = 0.019945). The DASD demonstrated a 50% reduction in the contamination of the OHCWs wrists and a 30% reduction in chest contamination compared to HV plus LV. The DASD in conjunction with LV was more effective in reducing aerosol, droplets and splatter than HV plus LV.

## 1. Introduction

The dental environment is unique in the high risk it poses for the transmission of infectious agents [1,2]. Oral health care workers (OHCW) can potentially be exposed to numerous pathogens (such as viruses, bacteria and fungi) that are present in the oral cavity and respiratory tract of patients [3]. OHCWs are amongst the highest risk group for disease contamination by aerosols, droplets and splatter [4]. The origin of airborne contaminants in a dental setting could be in the form of saliva, dental instruments, patient respiratory sources and the oral cavity [5]. Water combined with compressed air produces aerosol, droplets and splatter, which become contaminated by the oral cavity [6]. Aerosol-generating procedures (AGPs) produce a mixture of aerosol, droplets and splatter containing blood and saliva with various microorganisms [7]. This creates a working environment with a high potential of disease transmission [8]. The literature has demonstrated numerous sources of aerosol, droplet and splatter production in the dental environment—such as ultrasonic scalers and high-speed air turbines [9,10]

Dental lasers are also considered aerosol-producing devices due to the generation of a plume during procedures [11]. During laser procedures, a high-efficiency particulate filtration respirator (N99/FFP3 respirator) with a filter efficiency of 99.75% at 0.1 μm has been recommended [11]. The literature has demonstrated that the aerosols, droplets and splatter produced in large quantities during daily dental procedures are contaminated with pathogens [12]. These contaminated aerosols and droplets can remain suspended for extended periods of time before entering the respiratory tract or settling on surfaces [13]. Aerosols generated during dental procedures may remain suspended in the air for several hours and can spread up to 3 m from the source [9]. Aerosolized particles generated by dental equipment range from aerosol to droplets to splatter (0.001 to 50 µm). Particle sizes influence the time of suspension and settling of these aerosolized particles. Particles greater than 100 µm can be classified as splatter and settle quickly on surrounding surfaces and the floor. Droplet particle sizes that are smaller than 50 µm remain suspended in the air for extended periods [14].

Studies have demonstrated that aerosolized particles of 1 µm consist of sufficient volume to harbor a variety of respiratory pathogens and allow disease transmission. This has been demonstrated when pathogen transmission with the measles virus (50–500 nm) [15], influenza virus (100 nm to 1 μm) [16] and Mycobacterium tuberculosis (1–3 μm) has been studied. The current SARS-CoV-2 coronavirus pandemic brought to the forefront the concern of infection spread and transmission in the dental setting [17]. The pandemic resulted in a nearly complete halt in dental treatment across the world due to various lockdown regulations. In Italy and many other countries, dental treatment was limited to urgency and emergency treatment that could not be postponed. Dentistry performed during the initial stages of the SARS-CoV-2 pandemic was highlighted by an Italian study. It was reported that 69.5% of dentists managed dental emergencies recognized by the American Dental Association. Further, 68.2% of the dentists reported a fear of contracting SARS-CoV-2 after treatments were performed [18]. The importance of reducing the exposure of OHCW and the dental environment to aerosols, droplets and splatter generated during dental procedures has now become of even greater concern. Routes of transmission of SARS-CoV-2 coronavirus in humans include contact transmission (by means of contact with oral, nasal and eye mucosal membranes) and direct transmission (by means of coughing, sneezing and droplet inhalation transmission) [13]. Patients that are SARS-CoV-2 coronavirus positive, however asymptomatic, will inevitably present to dental practices for treatment. Studies have demonstrated that these asymptomatic SARS-CoV-2 coronavirus patients, as well as those recovering from acute illness, continue to shed significant amounts of the virus [19]. Studies have also demonstrated that symptomatic and asymptomatic SARS-CoV-2 coronavirus-positive patients presented with very similar viral loads [20]. Thus, the potential for generated droplets and aerosolized particles from these patients during procedures are at high risk for contaminating the air and surfaces of the entire dental practice [13]. The aim should thus be to reduce the amount of aerosol, droplets and splatter to an absolute minimum during this SARS-Cov-2 coronavirus pandemic [5]. The aim of this study was to assess the efficacy of a novel dental aerosol suction device (DASD) in reducing aerosol, droplets and splatter contamination.

## 2. Materials and Methods

### 2.1. Experimental Setup

The experiment was carried out in a 16 m^2^ dental surgery. A simulated clinical scenario was created with the high-speed air turbine. The OHCW (SMVS) positioned the air turbine above the right mandibular molar (tooth 46, FDI World Dental Federation notation) of a live volunteer (RM). The simulated clinical scenario was defined as the dental bur in the high-speed air turbine, directed 1 mm away from the central fissure of tooth 46 of the volunteer (RM) for 5 min. This would simulate the time spent on a cavity preparation, based on authors reporting full crown preparations in six minutes [21]. The tooth of the volunteer never made contact with the diamond bur during the simulation. The assistant and independent researcher (NN) held the Dental Aerosol Suction Device (DASD), high-volume evacuation and low-volume saliva ejector as per the study design. This simulated clinical scenario facilitated the in vivo assessment of the novel Dental Aerosol Suction Device (DASD). The OHCW was positioned 40 cm from the volunteer oral cavity holding the high-speed air turbine in position. The assistant was 40 cm from the volunteer oral cavity holding the low-volume saliva ejector (LV) and other devices that were assessed in position.

### 2.2. Equipment Used

**Figure 1:**
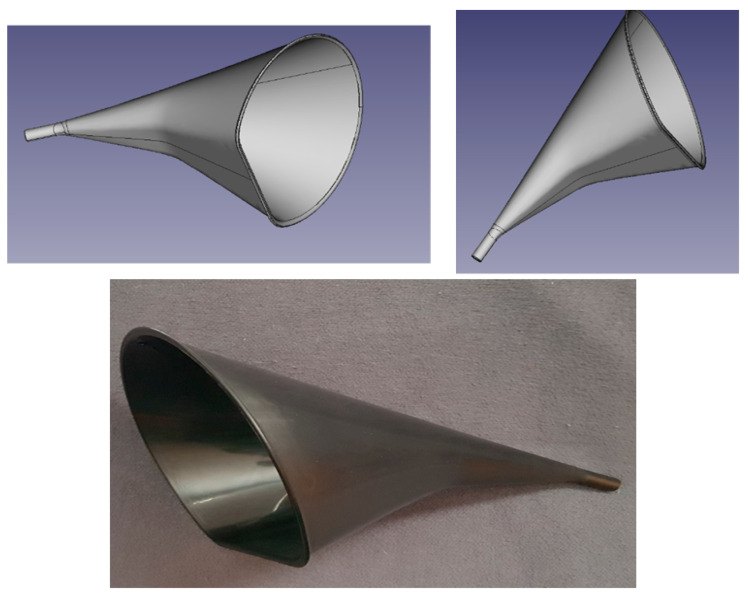
Dental Aerosol Suction Device (DASD).

The DASD device is patented in the United Kingdom under registration 6119833. The international design classification cover Class 24: Medical and laboratory equipment with subclass 02: Medical instruments, instruments and tools for laboratory use. The manufacturer ensures the CE marking. The DASD device is manufactured from a durable Nylon material under the ISO 13485. The DASD is autoclavable and capable of attaching to the high-volume evacuation adapter of the dental unit. The DASD device does not require additional motors or power sources to operate. The DASD design optimizes the catchment area, ergonomics of holding the device and aerodynamics to optimize the suction volume when larger than 300 L/min. The equipment utilized in this study included: DASD: Dental aerosol suction device (The University of the Western Cape, Cape Town, South Africa) (Figure 1); low-volume saliva ejector (LV) (Removable 6IN clear 22810148, Henry Schein, Johannesburg, South Africa); 11 mm high-volume straight evacuation tip (HV) (Saliva Ejec white 11 mm 078110, Henry Schein, Johannesburg, South Africa); Aspijet7 mobile suction unit at 400 L/min suction when the low-volume and high-volume suction adapters are open in full (Cattani ESAM, Worcestershire, UK) Durr suction vacuum and airflow rate volume gauge (Durr Dental SE, Bietigheim-Bissingen, Germany); high-speed air turbine (Pana-Max PLUS, NSK, Kanuma, Japan) (340,000 rpm), fitted with an inverted cone diamond bur (FG320R-5, Kerr, Brea, CA, USA) directed at the central fissure of the tooth 46 molar. Coolant flow rates were adjusted to 15 mL/min for the high-speed air turbine.

### 2.3. Measurement Method

**Figure 2:**
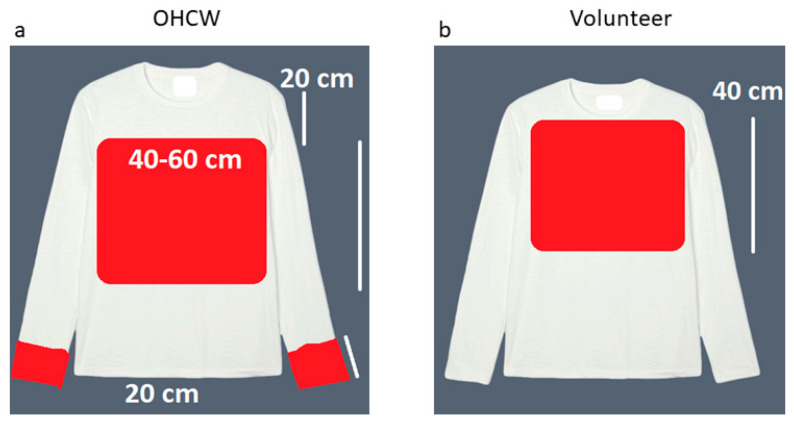
Long-sleeved fluid-resistant protective gown surfaces assessed for contamination (**a**) OHCW chest and wrists and (**b**) volunteer chest.

The long-sleeved fluid-resistant protective gowns were assessed under 1.75 × magnification (Start International, Dallas, TX, USA) for red contamination from the aerosol, droplets and splatter that occurred during the simulated clinical scenario. This clinical scenario was replicated in triplicate, each time with new long-sleeved fluid-resistant protective gowns. The areas contaminated from aerosol, droplets and splatter presented as red contaminated areas on the fluid-resistant protective gowns and were quantified under magnification in cm^2^ from an overlaid A4 paper size clear transparency with 1 cm^2^ blocks. The cm^2^ blocks were counted until a 1 cm^2^ block with no visible contamination was encountered—demarcating the end of contamination. The red color of the coolant was derived from red concentrate added in a concentration of 50 mL to 1000 mL distilled water coolant. The water line was purged with this prepared coolant prior to starting the in vivo study. The surfaces evaluated for contamination from aerosol, droplets and splatter were the chest of the volunteer (RM), the wrists and the chest of the OHCW (SMVS) (Figure 2). The chest of the OHCW was evaluated from a position 20 cm below the collar, as this is where the protective shield ends. The assessment area for the wrists was demarcated as the circumferential area of 20 cm (of the protective gown sleeves). The chest of the volunteer was demarcated as 40 cm starting at the collar. The width of the chest area was 30 cm. The test groups consisted of the control group (LV): low-volume saliva ejector on the low-volume evacuation adapter alone in the left lingual fossa; the high-volume evacuation (HV) consisted of the conventional high-volume evacuation 11 mm diameter tip 1 cm away from the high-speed air turbine head attached to the high-volume adapter and the LV as described; followed by the Dental Aerosol Suction Device (DASD) attached to the high-volume suction adapter with the LV as described. The DASD device is held by the assistant 10 cm away from the corner of the mouth in the 5 O’clock position. The air conditioner system in the dental surgery was off, and no natural ventilation was present with doors and windows closed.

### 2.4. Statistical Analysis

The data were analyzed using R Core Team (2013); (R: A language and environment for statistical computing. R Foundation for Statistical Computing, Vienna, Austria). The mean and standard deviation was calculated for the aerosol, droplets and splatter produced during the simulated clinical scenarios with the various aerosol reducing equipment. The Kruskal–Wallis rank-sum test for multiple independent samples was used. The Tukey–Kramer (Nemenyi) post-hoc pairwise multiple comparison tests were conducted to assess the significant differences. A *p*-value ≤ 0.05 was considered statistically significant.

## 3. Results

The Kruskal–Wallis rank-sum test for multiple independent samples resulted in a *p*-value = 0.027324. The post-hoc pairwise multiple comparison test was conducted to discern which of the pairs have significant differences. The input data reveals no ties in the ranks; therefore, *p*-value adjustments were not applicable. Tukey–Kramer (Nemenyi) *p*-values indicated a significant difference between the contamination of the volunteer chest, oral health care workers’ chest and wrists when LV alone was used compared to the DASD plus LV (Table 1). There was no significant difference between the LV alone and HV plus LV (*p* = 0.372059).

## 4. Discussion

The oral cavity harbors multiple pathogens with the potential of infecting OHCW during aerosol-generating dental procedures [22]. An analysis performed by the Alberta Federation of Labor established that OHCW is listed amongst the top 100 occupations with the highest risk of SARS-CoV-2 coronavirus exposure. This analysis also stratified exposure risk as follows: dental technologists 62.5% risk, dental assistants 97.5% risk, dentists 97% risk and dental hygienists and therapists carry 100% risk [23]. The literature currently does not include an assortment of in vivo data on the effectiveness of current devices to reduce aerosol, droplet and splatter contamination in dentistry [5]. Low-volume saliva ejectors (LV) alone have been deemed insufficient for aerosol-generating procedures in dental practice. Studies evaluating LV alone in the simulated clinical scenario detected very high concentrations of ultrafine aerosol particles (<10 µm) [21].

Currently, the use of high-volume evacuation and rubber dams are techniques aimed at minimizing microbial loaded dental aerosol and droplet contamination [12]. Studies have concluded that the use of rubber dams does reduce the microbial contamination of the operator and surrounding dental environment [24]. However, rubber dams have also been demonstrated to be associated with increased contamination of sterile head scarfs, as the rubber dam increases the average particle size [25]. Han et al. (2021) concluded that more studies are required in order to test the efficacy of aerosol, droplet and splatter reduction with dental high-volume evacuation devices [7]. Some authors have suggested that extra-oral motor-driven suction devices are good tools to reduce aerosol during dental treatment [8,26,27]. Study data regarding a reduction in aerosol particle movement demonstrated it in a dental setting when using a motor-driven extra-oral suction device [17]. The Isolite^®^ device is an example of an extra-oral suction device. A study evaluating the Isolite^®^ device assessed variables such as plaque, saliva, patient and operator position. This study demonstrated no significant difference in aerosol, droplet and splatter with regards to colony-forming units with the Isolite^®^ device, compared to low-volume saliva ejector alone [28].

Air purifiers have also been utilized in an attempt to reduce aerosol spread. The use of air purifiers was demonstrated to be insignificant in clinical settings where the cubicles are open, and multiple dental chairs are positioned in close proximity [17]. Natural air ventilation has been shown to assist in the dissemination of particulates away from areas where it would have settled. A study demonstrated that 1 m from the OHCW and 0.5 m from the saliva evacuation unit, a greater volume of particulate settled when low-volume saliva ejector was used with natural ventilation, compared to low-volume saliva ejector alone [29]. This study evaluated the aerosol, droplet and splatter reduction that could be achieved with a relatively inexpensive device (DASD). This device is not motor driven, does not require an additional power source and can be directly connected to the dental chair high-volume evacuation adapter. A study discussing the design of aerosol suction devices concluded that an optimal device size and shape were required to ensure aerosol reduction [5]. The DASD device is unique in its design and large catchment area, which optimizes the available high-volume suction capacity of the dental unit (above 300 L/min).

**Figure 3:**
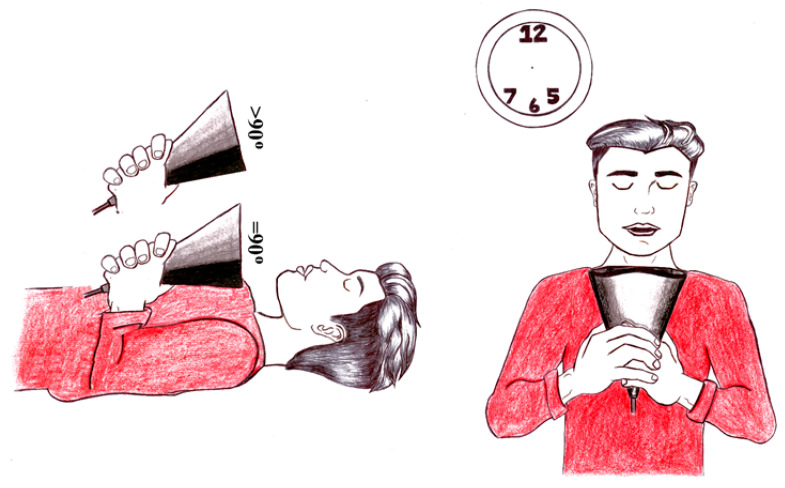
Positioning possibilities of the Dental Aerosol Suction Device.

Larger, more expensive extra-oral high-volume suction devices have integrated suction motors and air filters. A recent study evaluated an extra-oral high-volume device positioned superior-perpendicular to the mannequin’s oral cavity. This extra-oral device achieved a significant reduction in operator contamination compared to high-volume evacuation [30]. A study identified the drilling side and corresponding location to be the ideal position for high-volume evacuation tips [21]. An advantage of the DASD design and large catchment area is that the device positioning for optimal aerosol and splatter reduction is achievable from multiple positions. The DASD device can be positioned on the chest of the patient anterior to the oral cavity (termed anterior-perpendicular in the 6 O’clock position) and laterally (5 and 7 O’clock positions) (Figure 3). This allows versatile positioning of the DASD device, which does not interfere with the working field of the operator and the position of the overhead light. The DASD is recommended to be positioned 10 to 15 cm from the working area. This provides the advantage of not impeding the OHCW’s field of vision, compared to high-volume suction that needs to be positioned up to 1 cm adjacent to the working field.

A recent study assessed and compared commercially available high-volume evacuation and an extra-oral motor-driven vacuum system. The authors concluded that the low-volume saliva ejector alone was not adequate during aerosol-generating procedures. The authors also demonstrated no significant difference regarding aerosol contamination of OHCW when low-volume saliva ejector or high-volume evacuation was combined with a motor-driven extra-oral vacuum system [5]. The current DASD study demonstrated a 53% reduction in aerosol, droplet and splatter contamination of an OHCW when a high-volume evacuation was used in conjunction with a low-volume saliva ejector, compared to a low-volume saliva ejector alone. The DASD demonstrated a 62% reduction in aerosol, droplet and splatter contamination of an OHCW when used in conjunction with a low-volume saliva ejector. The aerosol, droplet and splatter contamination of the chest of the patient and the OHCW is a concerning factor. Studies have demonstrated that aerosols, droplets and splatters have been identified as far as 1.2 m from the source [7]. A study demonstrated that high-volume evacuation markedly reduced aerosol and droplet at the patient’s position of the oral cavity and at the level of the clinician when compared to low-volume saliva ejectors [5]. Contamination of the chest and wrist of OHCW during aerosol-generating procedures carries a high risk of cross-contamination and self-inoculation. The DASD achieved a 50% reduction in the contamination of the OHCW wrists and demonstrated a 30% reduction in the contamination of the OHCWs chest (DASD plus LV), compared to high-volume evacuation plus LV.

A study evaluating high-volume evacuation with high-volume evacuation plus extra oral motorized vacuum system achieved a 50% reduction in wrist contamination [30]. The DASD device plus LV achieved the same results utilizing the chair suction alone (at ≥300 L/min), without the need for additional motorized devices and power sources. Studies have demonstrated the presence of SARS-CoV-2 in the aerosol produced during patient exhalation [31,32]. During aerosol generation, the high-volume evacuation tip only operates for limited time frames during close approximation to the tooth during procedures, which reduces the efficacy of minimizing aerosols exhaled by the patient [5]. The DASD has the advantage of a large catchment area and can be placed statically to continuously eliminate aerosols produced during patient exhalation from the 6 O’clock position.

## 5. Conclusions

SARS-CoV-2 coronavirus has brought the importance of infection control and aerosol reduction in dentistry to the forefront. However, the importance of creating a safe working environment in dentistry should be a priority at all times. All OHCW should be practicing in a manner that ensures infection control (with a focus on the control of aerosol, droplets and splatter) and strictly adhere to disinfection protocols.

The DASD device presents as a cost-effective option to reduce aerosol, droplets and splatter, compared to current more expensive extra-oral evacuation systems. Greater levels of aerosol, droplets and splatter reduction to the wrists of the OHCW, as well as the chest of the OHCW and volunteer, was achieved with this device. The DASD enables aerosol, droplets and splatter reduction from multiple positions without impeding OHCW visibility and accessibility to the oral cavity.

## 6. Limitations

The contamination of the assistant was not recorded in this study. The dental bur positioned 1 cm away from the tooth fissure might not cover all clinical scenarios of cavity preparation, as the occlusal aspect of the tooth is essentially a flat, smooth surface. In a cavity preparation, the coolant would be directed into and then out of the cavity, possibly at a different trajectory compared to the simulated clinical scenario. Contamination was quantified in cm^2^ blocks, irrespective of the degree of red contamination per cm^2^. The addition of the red concentrate to the distilled water (similar to studies utilizing sodium fluorescein) could potentially alter the water tension, flow of the red coolant and aerosol, droplet and splatter sizes. The change in water tension with the red concentrate was not compared to distilled water prior to the study. A recommendation for future studies includes the assessment of aerosol generation during procedures, such as tooth polishing, surgical interventions and ultrasonic scaling. Further research could be conducted with DASD and a low-volume saliva ejector to indicate the efficacy of the device in those procedures with an electronic particle sensor.

## Figures and Tables

**Table 1:** Collection of aerosol and splatter on different areas of the OHCW and volunteer.

Device Assessed	Volunteer Chest (cm^2^)	OHCW Chest below Shield (cm^2^)	OHCW Wrists (cm^2^)
LV only	105 (±6.02)	357 (±4)	118 (±4)
HV + LV	55 (±5)	192 (±4.58)	71 (±2.64)
DASD + LV	25 (±2) *	133 (±2) *	35 (±1) *

*, *p* = 0.019945.

## Data Availability

Available upon request.
