# Correction: Noordien et al. In Vivo Study of Aerosol, Droplets and Splatter Reduction in Dentistry. *Viruses* 2021, *13*, 1928

**DOI:** 10.3390/v15081669

**Published:** 2023-07-31

**Authors:** Naeemah Noordien, Suné Mulder-van Staden, Riaan Mulder

**Affiliations:** 1 Paediatric Dentistry, The University of the Western Cape, Cape Town 7530, South Africa; nnoordien@uwc.ac.za; 2 Oral Medicine, Periodontology and Implantology Department, The University of the Western Cape, Cape Town 7530, South Africa; 3 Restorative Dentistry, The University of the Western Cape, Cape Town 7530, South Africa; rmulder@uwc.ac.za

## Text Correction

There was an error of omission in the original publication [1], in the Conflicts of Interest section: “The author N.N. declares no conflict of interest. R.M. and S.M.-v.S. are the inventors 11 of the DASD device.”

A correction has been made to the Conflicts of Interest section. The revised text is: 

“The author N.N. declares no conflict of interest as an independent researcher. R.M. and S.M.-v.S. are the inventors of the DASD device, full-time staff members of the University of the Western Cape and joint patent holders with the University of the Western Cape. The DASD device was licensed for manufacture and sale to Amplitude Consulting PTY (LTD) between October 2021 and December 2022. R.M. and S.M.-v.S. are shareholders of Amplitude Consulting PTY (LTD).”

The authors state that the scientific conclusions are unaffected. This correction was approved by the Academic Editor. The original publication has also been updated.
